# Specific AAV2/PHP.eB-mediated gene transduction of CA2 pyramidal cells via injection into the lateral ventricle

**DOI:** 10.1038/s41598-022-27372-8

**Published:** 2023-01-06

**Authors:** Kazuki Okamoto, Yuji Kamikubo, Kenta Yamauchi, Shinichiro Okamoto, Megumu Takahashi, Yoko Ishida, Masato Koike, Yuji Ikegaya, Takashi Sakurai, Hiroyuki Hioki

**Affiliations:** 1 Department of Neuroanatomy, Juntendo University Graduate School of Medicine, Bunkyo-Ku, Tokyo, 113-8421 Japan; 2 Department of Cell Biology and Neuroscience, Juntendo University Graduate School of Medicine, Bunkyo-Ku, Tokyo, 113-8421 Japan; 3 Juntendo Advanced Research Institute for Health Science, Juntendo University, Bunkyo-Ku, Tokyo, 113-8421 Japan; 4 Department of Cellular and Molecular Pharmacology, Juntendo University Graduate School of Medicine, Bunkyo-Ku, Tokyo, 113-8421 Japan; 5 Department of Neuroscience, Graduate School of Medicine, Kyoto University, Kyoto, Kyoto 606-8501 Japan; 6 Research Fellow of Japan Society for the Promotion of Science (JSPS), Chiyoda-ku, Tokyo, 102-0083 Japan; 7 Laboratory of Chemical Pharmacology, Graduate School of Pharmaceutical Sciences, The University of Tokyo, Bunkyo‐ku, Tokyo, 113‐0033 Japan; 8 Center for Information and Neural Networks, National Institute of Information and Communications Technology, Suita, Osaka 565-0871 Japan; 9 Institute for AI and Beyond, The University of Tokyo, Bunkyo‐ku, Tokyo, 113‐0033 Japan; 10 Department of Multi-Scale Brain Structure Imaging, Juntendo University Graduate School of Medicine, Bunkyo-Ku, Tokyo, 113-8421 Japan

**Keywords:** Neuroscience, Neural circuits

## Abstract

Given its limited accessibility, the CA2 area has been less investigated compared to other subregions of the hippocampus. While the development of transgenic mice expressing Cre recombinase in the CA2 has revealed unique features of this area, the use of mouse lines has several limitations, such as lack of specificity. Therefore, a specific gene delivery system is required. Here, we confirmed that the AAV-PHP.eB capsid preferably infected CA2 pyramidal cells following retro-orbital injection and demonstrated that the specificity was substantially higher after injection into the lateral ventricle. In addition, a tropism for the CA2 area was observed in organotypic slice cultures. Combined injection into the lateral ventricle and stereotaxic injection into the CA2 area specifically introduced the transgene into CA2 pyramidal cells, enabling us to perform targeted patch-clamp recordings and optogenetic manipulation. These results suggest that AAV-PHP.eB is a versatile tool for specific gene transduction in CA2 pyramidal cells.

## Introduction

The hippocampal CA2 region is an anatomically small area located between the CA3 and CA1 regions. Due to the restricted size, it has been less investigated than the other hippocampal areas, both molecularly and physiologically. The CA2 region was first described by Lorente de Nó based on its cytoarchitecture^1^, and it has recently been identified based on its molecular expression profiles, including that of regulator of G-protein signaling 14 (RGS14)^2–7^. Using transgenic mouse lines expressing Cre recombinase predominantly in CA2 pyramidal cells, the unique features of this region, such as processing of social memory^8^, dynamic regulation of the hippocampal network^9^, and coding of temporal information^10^ have been clarified. Now the CA2 Cre lines are becoming the essential tools for the study targeting the CA2 region. In the present study, we proposed an alternate approach for gene transfer to the CA2 region with adeno-associated virus (AAV) vector injections.

AAV vectors are among the most-favored vehicles for gene transfer to the nervous system. They have different cell affinities depending on their capsid serotypes^11^. AAV-PHP.eB, a capsid variant of AAV serotype 9, was originally developed to cross the blood–brain barrier (BBB)^12^; intravenous infusion of AAV-PHP.eB results in efficient gene delivery to the whole brain. Yet, the transduction is not so homogenous that it preferentially infects some brain regions, including the CA2 area^12,13^.

Here, we took advantage of the tropism of AAV-PHP.eB for the CA2 region via lateral ventricle (LV) injections and developed an alternative method to label CA2 pyramidal cells using the capsid. We demonstrate the specific gene transfer to CA2 pyramidal cells by injection of this vector into the LV of mice and optogenetic manipulation of CA2 pyramidal cells. In addition, the CA2 region was preferentially infected by AAV-PHP.eB in organotypic slice cultures. Thus, the AAV vector pseudotyped with PHP.eB capsid is a versatile genetic tool for the specific labeling and manipulation of CA2 pyramidal cells.

## Results

### Preference of AAV-PHP.eB for hippocampal CA2 pyramidal cells via intravenous injection

**Figure 1:**
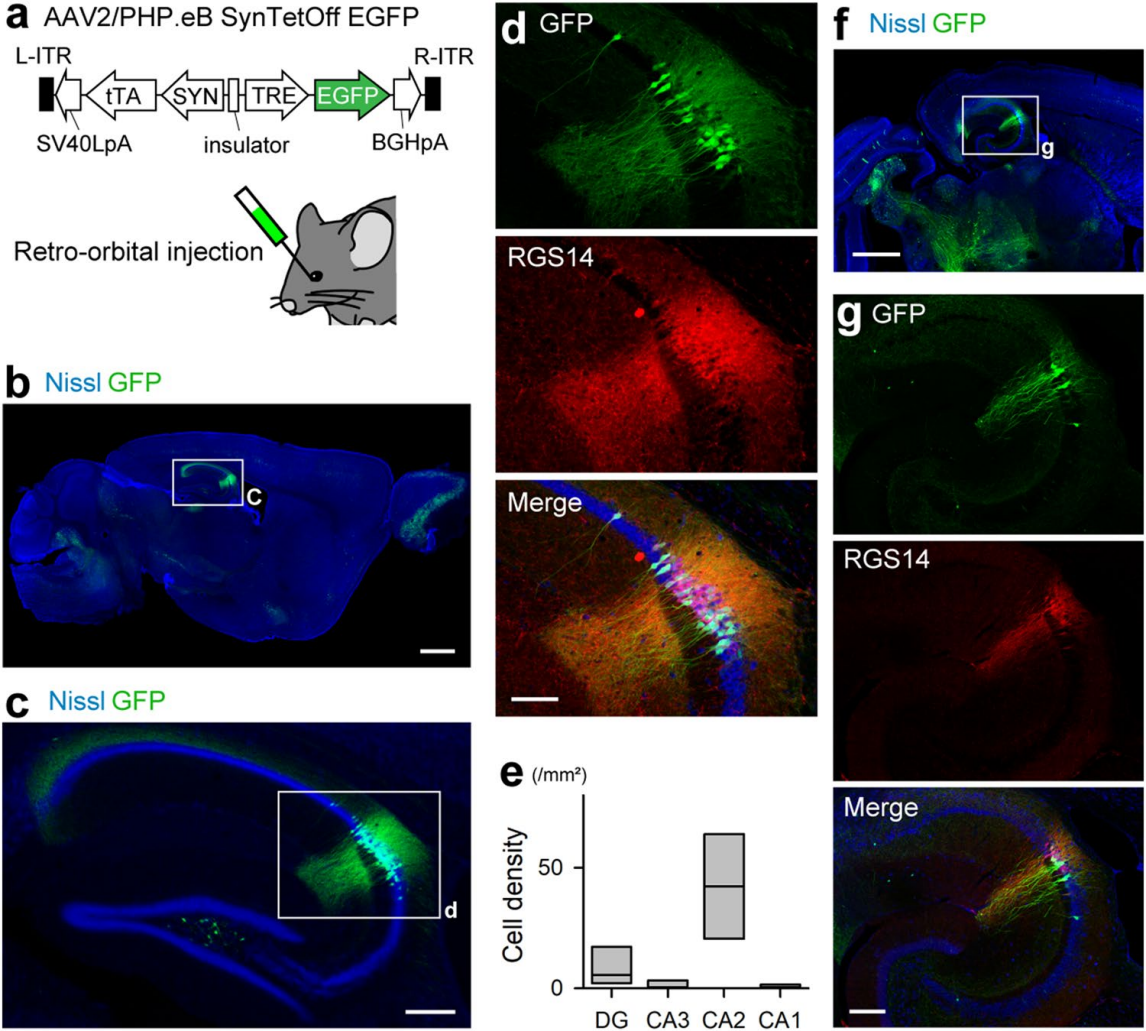
Preferential expression of GFP for CA2 pyramidal cells with AAV-PHP.eB capsid. (**a**) AAV2/PHP.eB-SynTetOff-EGFP vector (top) and a schematic view of the retro-orbital injection (bottom). (**b**) GFP expression in a sagittal section after retro-orbital injection of the vector. Scale bar, 1 mm. (**c**) Enlarged view of the hippocampus in (**b**). Scale bar, 200 μm. (**d**) Enlarged view of the CA2 region in (**c**). Scale bar, 100 μm. (**e**) Distribution of GFP-expressing cells in the hippocampal subregions. The rectangles indicate the means and distributions of 25% and 75% (*n* = 4 sections from 4 mice, *H* = 11.5, *df* = 3, *P* = 9.1 × 10^−3^, Kruskal–Wallis test). (**f**) Same as (**b**), but in a horizontal section at the level of the intermediate hippocampus. Scale bar, 1 mm. (**g**) Enlarged view of rectangle in (**f**). Scale bar, 200 μm.

AAV vectors packaged with PHP.eB capsid can cross the BBB^12^. We infused AAV2/PHP.eB-SynTetOff-EGFP (1.4 × 10^11^ genome copies (gc)/mouse) by retro-orbital injection, which is a method of intravenous injection (Fig. 1a and S1a). One week after the injection, GFP expression was found widely throughout the brain, whereas strong GFP signals were observed in certain brain regions, such as the olfactory bulb, cerebellar nucleus, and hippocampus (Fig. 1b). The expression pattern was similar to that observed using the lateral tail vein injection, another intravenous injection method^14^ (Fig. S1b). However, the vector equipped with the Tet-Off system displayed different GFP expression pattern with other promoters, such as CAG and synapsin I (SYN)^15^ (Fig. S2). Remarkably, in the hippocampus, GFP-expressing cells were predominantly localized in the CA2 region (Fig. 1c). Most GFP-expressing cells (80 ± 14%) showed the immunoreactivity for RGS14, a specific marker for CA2 pyramidal cells (Fig. 1d)^2,3^. Similar results were obtained with other markers, Purkinje cell protein 4 (PCP4) and striatal-enriched protein tyrosine phosphatase (STEP) (PCP4, 91 ± 8.4%; STEP, 88 ± 12%; *n* = 3 sections from 3 mice; Fig. S3). Conversely, approximately 90% of RGS14-immunoreactive cells expressed GFP (87 ± 1.3%; *n* = 3 sections from 3 mice). Compared to that in the other hippocampal subregions, the CA2 area had a significantly larger number of GFP-expressing cells (dentate gyrus, 8.3 ± 8.4 cells/mm^2^; CA3, 1.1 ± 2.2 cells/mm^2^; CA2, 42 ± 23 cells/mm^2^; CA1, 0.56 ± 1.1 cells/mm^2^; Fig. 1e). In addition, GFP was highly expressed in the intermediate hippocampus^16^, colocalizing with RGS14 (Fig. 1f and g). These results are consistent with the previous reports that AAV-PHP.eB has the strong tropism for the CA2 region^13,17^, and further indicate infection preference for pyramidal cells within the CA2 region.

### Restricted infection of CA2 pyramidal cells via injection into the LV

**Figure 2:**
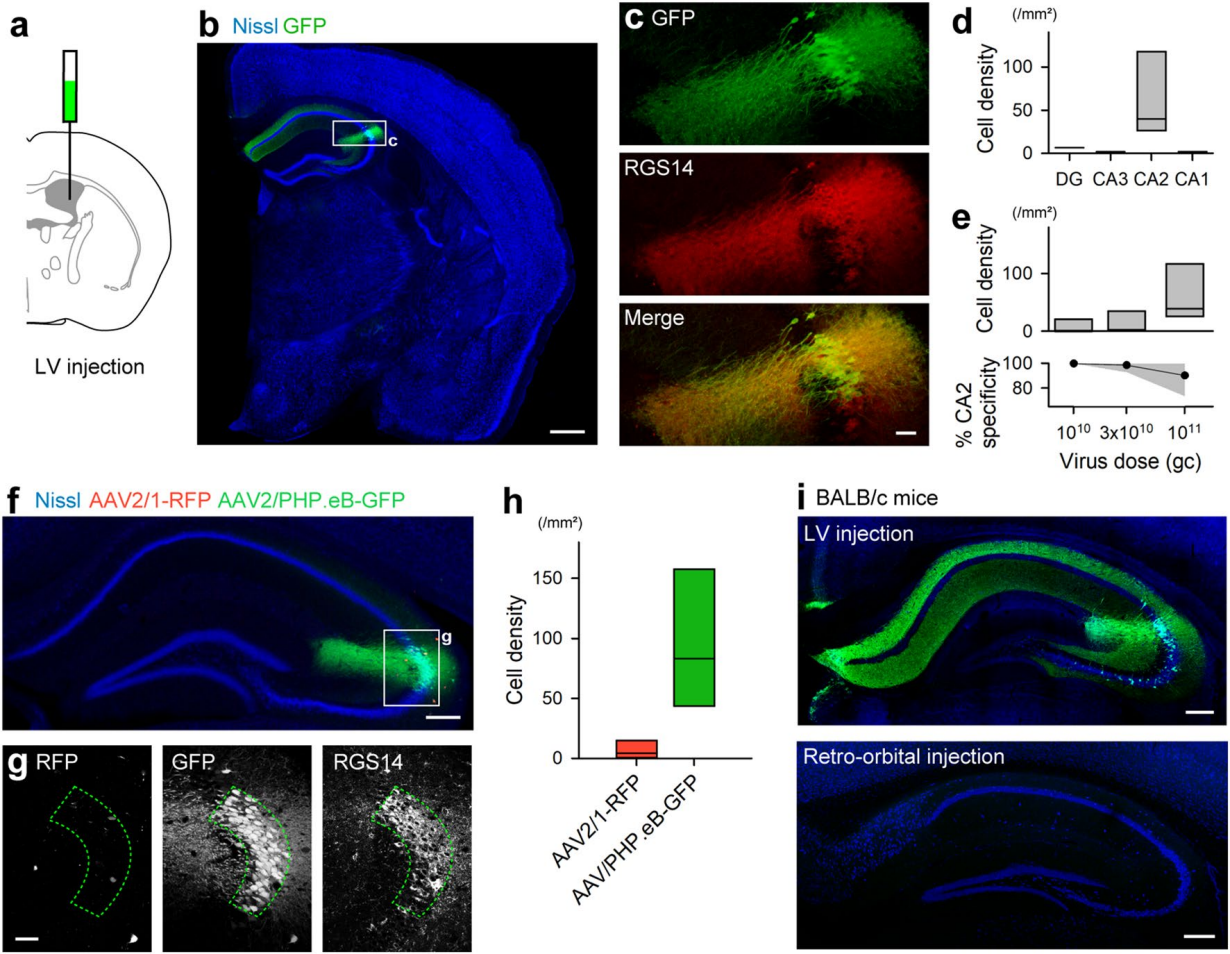
Restricted infection of CA2 pyramidal cells via injection into the LV. (**a**) A schematic view of LV injection. (**b**) GFP expression after LV injection of the AAV2/PHP.eB-SynTetOff-GFP vector. Scale bar, 500 μm. (**c**) Enlarged view of the CA2 region in (**b**). Scale bar, 50 μm. (**d**) Density of GFP-expressing cells in the hippocampal subregions. The lines and rectangle indicate the means and distributions of 25% and 75%, respectively (*n* = 5 sections from 5 mice, *H* = 13.7, *df* = 3, *P* = 3.4 × 10^−3^, Kruskal–Wallis test). (**e**) (Top) Virus dose-dependent increase in the infected cell density in the CA2. The rectangles indicate the means and distributions of 25% and 75% (*n* = 4–6 sections from 4 to 6 mice). (Bottom) Specificity for infection of CA2 pyramidal cells. Gray area indicates 95% confidence interval (*n* = 4–6 sections from 4 to 6 mice, *F* = 4.36, *df* = 2, *P* = 0.038, one-way analysis of variance (ANOVA)). (**f**) LV injection of AAV2/1-SynTetOff-mRFP1 and AAV2/PHP.eB-SynTetOff-EGFP. Scale bar, 200 μm. (**g**) Enlarged view of the CA2 area in (**f**). Scale bar, 50 μm. (**h**) Cell density of RFP- or GFPexpressing cells in the CA2 area. The rectangles indicate the means and distributions of 25% and 75% (*n* = 4 sections from 4 mice, *t* = 2.78, *df* = 6, *P* = 0.032, two-tailed unpaired Student’s *t*-test). (**i**) LV (top) or retro-orbital (bottom) injection of AAV2/PHP.eB-SynTetOff-EGFP in BALB/c mice. Scale bar, 200 μm.

To enhance the specificity of gene transduction in CA2 pyramidal cells, we injected the virus (1 × 10^11^ gc/mouse) into the bilateral LV (Fig. 2a). One week after the injection, GFP expression was observed exclusively in the hippocampus, especially in the CA2 region (Fig. 2b). There was no expression outside the hippocampus such as the olfactory bulb and cerebellar nucleus (Fig. S4a), except for brain regions surrounding the LV (Fig. S4b). Almost all GFP-expressing cells (90 ± 14%) in the hippocampus were immunoreactive for RGS14 (Fig. 2c), and the specificity of LV injection was higher than that of intravenous injection (80 ± 14%). The number of GFP-expressing cells was significantly higher in the CA2 region than that in the other regions of the hippocampus (dentate gyrus, 1.3 ± 3.0 cells/mm^2^; CA3, 0.44 ± 0.99 cells/mm^2^; CA2, 64 ± 56 cells/mm^2^; CA1, 0.44 ± 0.99 cells/mm^2^; Fig. 2d).

We then investigated the changes in the number of infected cells and specificity for RGS14 depending on the amount of virus particles injected into the bilateral LV. Increasing the virus dose increased the number of infected cells in the hippocampus (1.0 × 10^10^ gc, 7.2 ± 14.4 cells/mm^2^; 3.0 × 10^10^ gc, 14 ± 19 cells/mm^2^; 1.0 × 10^11^ gc, 66 ± 53 cells/mm^2^; Fig. 2e, top and Fig. S4c), whereas it decreased the overlap of GFP expression with RGS14 immunoreactivity (1.0 × 10^10^ gc, 100 ± 0%; 3.0 × 10^10^ gc, 99 ± 2.7%; 1.0 × 10^11^ gc, 90 ± 14%; Fig. 2e, bottom). In addition, we performed unilateral LV injection and observed that GFP expression was much higher in the ipsilateral side than that in the contralateral side (Fig. S4d).

Crossing the cerebrospinal fluid (CSF)-brain barrier was likely to be specific for AAV-PHP.eB. To verify the property, we injected a mixture of AAV2/1-SynTetOff-mRFP1 and AAV2/PHP.eB-SynTetOff-EGFP into the LV (Fig. 2f and g). While GFP signals were observed specifically in CA2 pyramidal cells, almost no RFP expression was detected in the hippocampus (Fig. 2f–h). In addition, we directly injected the mixture into the CA2 region. In both serotypes, infection was observed not only in the CA2 but also in the CA3 (Fig. S5), indicating that direct injection into the hippocampal parenchyma has no selectivity for the CA2. We further tested whether AAV-PHP.eB can cross the CSF-brain barrier in mouse strains other than C57BL/6J; AAV-PHP.eB is known to cross the BBB in C57BL/6J mice, but not in other strains, such as BALB/c and B6C3^13,17–19^. One week after the injection of AAV2/PHP.eB-SynTetOff-EGFP into the LV of BALB/c mice, GFP-expressing cells were found specifically in the CA2 region (Fig. 2i, top). In contrast, no signal was observed in the brain upon injection into the retro-orbital sinus, as reported previously (Fig. 2i, bottom)^18^. Collectively, these results indicate that LV injection of an AAV vector with PHP.eB enables specific labeling of CA2 pyramidal cells not only in C57BL/6J but also in BALB/c mice, suggesting that the tropism via LV injection can be leveraged in other rodents, independent of the BBB permeability.

### Specific gene delivery to the CA2 region in organotypic slice cultures using AAV-PHP.eB

We investigated whether AAV-PHP.eB had an infection preference for the CA2 region in rat hippocampal slice cultures, which preserve neuronal networks ex vivo and are suitable to study network functions^20,21^. Although the CA2 region is known to develop in these cultures^22^, it is not yet fully organized at postnatal days (P)7–8, the age at which we prepared slices^2,23^. Thus, slices must be incubated for at least one week to conduct AAV infection experiments in the CA2 region. As virus vectors become less infectious in slice cultures over time, viruses should be applied within 3 days in vitro (DIV)^24^.

**Figure 3:**
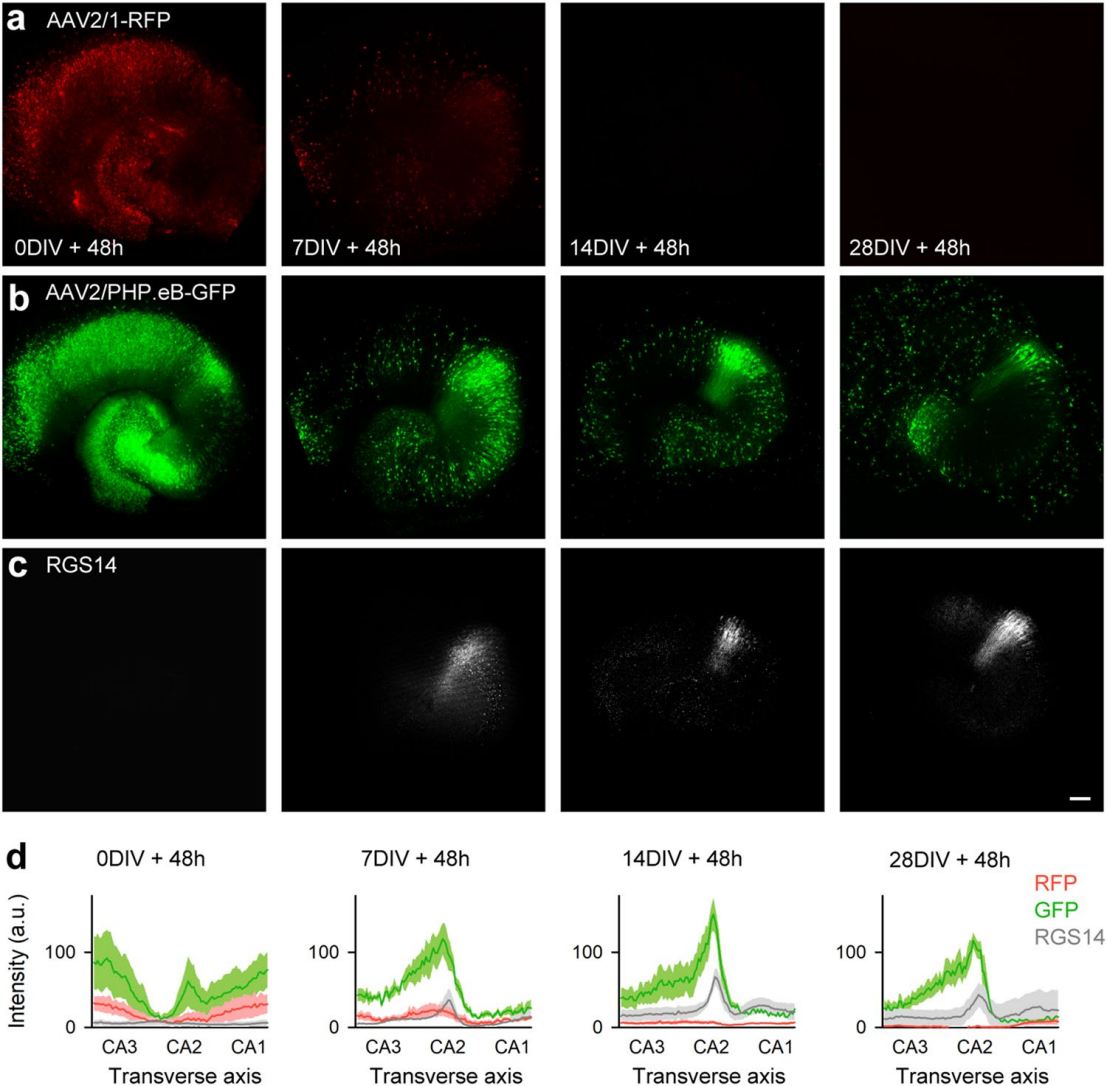
AAV2/PHP.eB vector preference for the CA2 region in ex vivo organotypic slice cultures. (**a**) RFP, (**b**) GFP, and (**c**) RGS14 protein distribution in hippocampal slice cultures. AAV2/1-SynTetOff-mRFP1 and AAV2/PHP.eB-SynTetOff-EGFP were added to slice cultures at 0, 7, 14, and 28 DIV; RFP/GFP signals and RGS14 immunoreactivity were analyzed 48 h later. Scale bar, 200 μm. (**d**) Signal intensities of RFP (red), GFP (green), and RGS14 (gray) at each time point along the transverse axis of cultured hippocampal slices. Red, green, and gray areas indicate standard errors (*n* = 5 slices from 5 rats).

We chose four different time points (0, 7, 14, and 28 DIV) for infection. We added a mixture of AAV2/1-SynTetOff-mRFP1 and AAV2/PHP.eB-SynTetOff-EGFP to the hippocampal slice cultures and examined both RFP and GFP signals by confocal laser scanning microscopy 48 h later (Fig. 3a and b). In the virus application at 0 DIV, both RFP and GFP signals were broadly observed in the cultured hippocampus (Fig. 3a and b). The number of RFP-expressing cells drastically decreased in the slices with application at 7 DIV and almost disappeared in those applied at 14 DIV (Fig. 3a). GFP-expressing cells also decreased with cultivation time, but were still observed locally after virus application at 28 DIV (Fig. 3b). These results indicate that virus infection spreads throughout the slice cultures with both AAV2/1 and AAV2/PHP.eB immediately after slice preparation (0 DIV), while the infection was successful only with AAV2/PHP.eB after 7 DIV.

The expression of RGS14 also changed with the cultivation time (Fig. 3c), similar to that in vivo^2^. Based on fluorescent Nissl staining and RGS14 immunoreactivity, we determined the location of the putative CA2 region and the transverse axis along with the stratum pyramidale from CA3 to CA1 (Fig. S6). We calculated the immunoreactivities for RFP, GFP, and RGS14 according to the transverse axis. At 0 DIV application, there was no obvious tropism of either AAV2/1 or AAV2/PHP.eB (Fig. 3d). In contrast, after 7 DIV, we found strong peaks in GFP immunoreactivity in the putative CA2 area, whereas no peak was observed in RFP (Fig. 3d). We also found a weak peak in RGS14 immunoreactivity at 7 DIV and strong peaks at 14 and 28 DIV. These results suggest that AAV-PHP.eB has a preference for the CA2 area in organotypic slice cultures of rats.

### Specific gene transduction and manipulation of CA2 pyramidal cells with AAV dual infection

**Figure 4:**
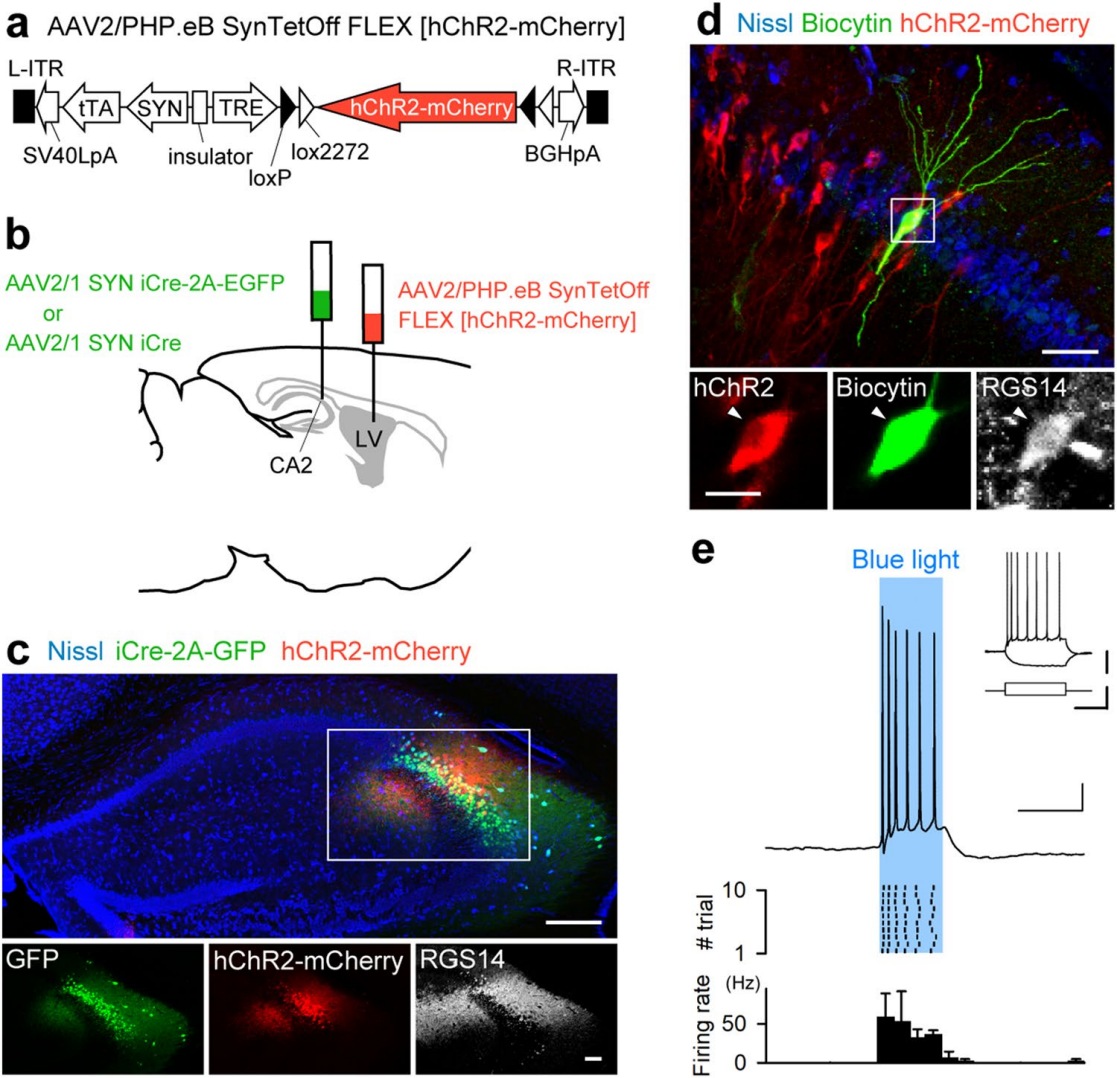
Specific gene transduction in CA2 pyramidal cells by AAV dual infection. (**a**) AAV2/PHP.eB-SynTetOff-FLEX-[hChR2-mCherry] vector. (**b**) Dual injection into the CA2 area (AAV2/1-SYN-iCre-2A-EGFP or AAV2/1-SYN-iCre) and the LV (AAV2/PHP.eB-SynTetOff-FLEX-[hChR2-mCherry]). (**c**) Expression of hChR2-mCherry and GFP in Nissl-stained hippocampus. Scale bars, 200 μm (top) and 100 μm (bottom). (**d**) Intracellular labeling of a hChR2-mCherry-expressing cell after targeted patch-clamp recording. Slice were immunostained for RGS14 and stained with a fluorescent Nissl. Top shows a merged view of biocytin (green), fluorescent Nissl (Blue), and hChR2-mCherry (Red). Bottom shows an enlarged image of the rectangle in the top. Arrowhead indicates the recorded neuron. Scale bars, 50 μm (top) and 20 μm (bottom). (**e**) (Top) Representative trace of the membrane responses to photostimulation at a duration of 100 ms. Scale bars, 100 ms (horizontal) and 20 mV (vertical). (Middle) Raster plot of spikes stimulated by blue-light illumination. (Inset) Membrane potential evoked by current injection into the same neuron. Scale bars, 100 ms (horizontal), 20 mV, and 400 pA (vertical). (Bottom) Firing rate was increased during blue-light illumination in recorded 4 cells from 3 mice.

We performed whole-cell patch-clamp recording on CA2 pyramidal cells visualized with AAV infection. Considering the decrease of CA2 selectivity by LV injections in a dose-dependent manner (Fig. 2e), a double injection approach was applied to obtain high selectivity while introducing transgene into many cells. We simultaneously injected AAV2/1-SYN-iCre-2A-GFP into the CA2 region and AAV2/PHP.eB-SynTetOff-FLEX-[hChR2-mCherry] into the LV (Fig. 4a and b); hChR2-mCherry is expressed via the Cre-dependent flip-excision (FLEX) system^25^. One week after the injection, GFP-expressing cells were observed to spread outside of the CA2 stratum pyramidale, while the expression of hChR2-mCherry was restricted to the region immunoreactive for RGS14 (Fig. 4c). This indicates that gene transduction specific to CA2 pyramidal cells is feasible using double infection via the Cre/loxP recombination system.

We prepared acute hippocampal slices one week after the dual injection using AAV2/1-SYN-iCre instead of AAV2/1-SYN-iCre-2A-GFP (Fig. 4b) and performed whole-cell patch-clamp recordings on an mCherry-expressing neuron. The recorded neuron was irradiated by blue light with a duration of 100 ms in current clamp mode. Depolarization and action potentials were recorded with 10 trains of light irradiation (Fig. 4e), indicating that hChR2 functioned properly in the recorded neuron. After the physiological recording, the neuron was intracellularly injected with biocytin and visualized post hoc using Alexa Flour 488 conjugated with streptavidin. The recorded neuron was located in the CA2 stratum pyramidale and showed the immunoreactivity for RGS14 (Fig. 4d). These results indicate that the here performed dual infection enables specific labeling of CA2 pyramidal cells and can be used for targeted patch-clamp recordings and optogenetics-mediated manipulation.

## Discussion

In the present study, we found that an AAV2/PHP.eB vector exhibits a strong preference for certain brain regions including the hippocampal region, olfactory bulb, and deep cerebellar nuclei following retro-orbital injection. In the hippocampal region, the AAV vector preferentially infected the CA2 pyramidal cells. We utilized this preference for selective transduction to the CA2 pyramidal cells following LV injection: 90% of the infected cells were CA2 pyramidal cells in the hippocampus. By combining LV injection with stereotaxic injection into the CA2 region, we succeeded in gene transfer specific to CA2 pyramidal cells and optogenetic manipulation of their activities. In addition, we demonstrate that an AAV2/PHP.eB vector preferentially infected CA2 pyramidal cells in hippocampal slice cultures. Therefore, AAV2/PHP.eB is an excellent genetic tool for gene transduction in CA2 pyramidal cells, both in vivo and ex vivo.

We demonstrated that LV injection of an AAV2/PHPe.B vector, but not AAV2/1, selectively infected the CA2 region across the CSF-brain barrier (Fig. 2). Using AAV serotype 9, a prototype capsid of PHP.eB, might yield similar results to that of the AAV2/PHPe.B vector. AAV9 exhibits neuronal tropism in the mouse hippocampus and enables high expression of the reporter protein^26^. An AAV9 vector efficiently crossed the CSF-brain barrier causing widespread cell transduction in the mouse brain^13^. Therefore, other serotypes, such as serotype 9, might be used for achieving selective infection of hippocampal CA2 neurons by LV injection.

After LV injection of the AAV2/PHP.eB vector, GFP expression was mainly observed in the dorsal CA2 region (Fig. 2). This was likely based on the point of injection, which was located in the anterior horn of the LV; the injected solution crossed the CSF-brain barrier dorsally with little diffusion^13^. In contrast, reporter gene expression was detected not only in the dorsal but also in the intermediate CA2 region following retro-orbital injection (Fig. 1f and g). Most CA2-specific genes, including *rgs14*, are not expressed in the ventral one-third of the hippocampus^5,27^. The part of the CA2 area in the intermediate hippocampus is often referred to as the “ventral CA2”^28^. Our results suggest that the AAV-PHP.eB vector also infects the intermediate (or ventral) CA2 area, with injection into the LV adjacent to the intermediate hippocampus.

There is a trade-off between specificity and the number of infected cells. Higher virus dose increases the number of infected cells, whereas the specificity for CA2 pyramidal cells decreases (Fig. 2e and S4c). At high dose (10^11^ gc), GFP expression was observed not only in CA2 pyramidal cells but also in stratum oriens interneurons and hilar interneurons. Low dose increases the specificity for CA2 pyramidal cells, but may not be sufficient for gene expression due to the lower number of virus copies in the infected neurons. Therefore, we used the AAV-SynTetOff platform, which increases the expression level 40 times compared to that of SYN promotor^15^ (Fig. S2). SYN promoter displayed weak expression in restricted brain regions including olfactory bulb, CA2 region, and cerebellum, whereas the SynTetOff platform dramatically increased the expression level of GFP in those brain regions. In contrast, strong GFP expression was observed throughout the brain with CAG promoter. These results suggest that the SynTetOff platform might affect the specificity.

We adopted an intersectional strategy with AAV dual infection to specifically transduce a large number of CA2 pyramidal cells (Fig. 4a), considering LV-peripheral expression (Fig. S4b). The intersectional approach has been applied to achieve accuracy in cell targeting via Cre/lox and/or Flp/FRT recombination systems using transgenic mice^29,30^, virus vectors^31,32^, and a combination of transgenic mice and virus vectors^33^. After injecting AAV2/PHP.eB-SynTetOff-FLEX-[hChR2-mCherry] into the LV and AAV2/1-SYN-iCre-2A-EGFP into the CA2 region locally, GFP expression was observed in a broad region of the hippocampus, including the CA2 area. In contrast, mCherry signals were localized exclusively in the CA2 region. Subsequently, we succeeded in CA2-targeted patch-clamp recording and optogenetic manipulation in ex vivo acute brain slices. The present intersectional approach could be useful for in vivo studies, such as the monitoring and/or manipulation of CA2 pyramidal cell activity.

Notably, gene transduction in CA2 pyramidal cells was achieved by adding the AAV2/PHP.eB vector to long-cultivated slice cultures (Fig. 3). In general, gene transfer into cultured slices using virus vectors should preferably be performed at least until 3 DIV^24^, since the infection efficiency decreases in a culture duration-dependent manner. In accordance with this view, gene transduction with an AAV2/1 vector in hippocampal slice cultures drastically decreased from 0 to 7 DIV. In contrast, gene transfer in slice cultures using an AAV2/PHP.eB vector was successful even at 28 DIV (Fig. 3), suggesting that this capsid can cross barrier-like structures. In the superficial layer of cultured slices, astrocytes accumulate tightly^34^, which may act as a barrier against the virus vector penetration. Astrocytes are components of BBB^35^ and CSF-brain barrier^36^.

In the present study, we demonstrate that the affinity of the AAV-PHP.eB capsid for CA2 pyramidal cells can be employed in a method of gene transfer into this region. This technique may contribute to the progress of unraveling the functional complexity of the CA2 region. Importantly, this method was applicable not only to the C57BL/6J but also to the BALB/c strain (Fig. 2i), although further investigation of CA2-specific gene transfer by LV injection using other strains is necessary. Using transgenic mouse lines that expresses Cre recombinase, such as *Amigo2*-*Cre*^8^ and *Amigo2*-*iCreERT2*, is the preferred way to specifically introduce a transgene into CA2 pyramidal cells^37^. However, in *Amigo2*-*Cre* mice, Cre recombinase is expressed not only in the CA2 region, but also sparsely in the hilus of the dentate gyrus, compromising specificity^8^. Hence, to avoid unspecific expression outside of the CA2 area, it is necessary to inject a Cre-dependent virus vector strictly into the CA2 region^38,39^. Our intersectional approach of LV injection can be applied to the CA2-Cre lines and achieve specific expression of transgene(s). It is a great advantage that gene transfer to CA2 pyramidal cells can be readily accomplished by injecting the AAV vector carrying the PHP.eB capsid into the LV; in addition, an experimental model similar to that using Cre-expressing transgenic mice can be constructed just by injecting a Cre-expressing AAV vector with the PHP.eB capsid into the LV. It is known that the CA2 region is an important circuit structure in the hippocampus, responsible for social memory function^8^ and the occurrence of sharp-wave ripples^39^; therefore, there is a growing need to establish CA2-specific gene transduction methods. We believe that the proposed method, which utilizes the properties of AAV-PHP.eB capsid, is versatile for gene transfer to CA2 pyramidal cells for labeling, activity monitoring, and manipulation.

## Methods

### Animals

All procedures involving animals were performed in accordance with the National Institutes of Health Guide for the Care and Use of Laboratory Animals. The experiments were approved by the Committees for Animal Care and Use and those for Recombinant DNA Study at Juntendo University (2021245, 2021246, and 2021248). Adult C57BL/6J mice (male and female; 8–10 weeks old; *n* = 17), juvenile C57BL/6J mice (male; P33–37; *n* = 3), adult BALB/c mice (male; 8–10 weeks old; *n* = 2), and pups of Sprague–Dawley rats (male and female; P6 of gestation; *n* = 9) were obtained from Nihon SLC. Animals were maintained under a 12-h light/dark cycle and had ad libitum access to food and water. All efforts were made to minimize animal suffering and the number of animals used. This study was carried out in compliance with the ARRIVE guidelines.

### Plasmid construction

For construction of pAAV2-SynTetOff-mRFP1, the sequence of an mRFP1 fragment (GenBank AF506027.1)^40^ was amplified by polymerase chain reaction (PCR; primer set P1/P2; Table S1). The EGFP sequence in pENTR1A-SV40LpA-tTAad-SYN-insulator-TRE-GFP-BGHpA^15^ was replaced with the PCR product using the *BamHI*/*MluI* sites, resulting in pENTR1A-SV40LpA-tTAad-SYN-insulator-TRE-mRFP1-BGHpA. The entry vector was converted to pAAV2-SynTetOff-mRFP1 with pAAV2-DEST(f)^15^ using LR clonase II (11791020; Thermo Fisher Scientific).

pAAV2-SynTetOff-hFLEX-[hChR2-mCherry] was constructed as follows: a polyadenylation signal derived from the bovine growth hormone gene (BGHpA; nucleotides 1771–1995 in GenBank AH009106.2) was derived from pAAV2-SynTetOff-GFP^15^ and inserted into the *MluI*/*NotI* sites of pBSIISK-hFLEX^15^, resulting in pBSIISK-hFLEX-BGHpA. The hChR2-mCherry fragment was amplified by PCR (primer set P3/P4) from pAAV-CaMKIIa-hChR2(H134R)-mCherry (#26975; Addgene), and inserted into the *EcoRI*/*SalI* sites of pBSIISK-hFLEX-BGHpA, resulting in pBSIISK-hFLEX-[hChR2-mCherry]-BGHpA. The hFLEX-[hChR2-mCherry]-BGHpA fragment was inserted into the *KpnI*/*SphI* sites of pAAV2-SynTetOff-BBS^41^ to generate pAAV2-SynTetOff-hFLEX-[hChR2-mCherry].

For construction of pAAV2-SYN-iCre-BGHpA, the oligonucleotide set (P5/P6) was annealed to form double-stranded DNA containing *HindIII*-*BglII*-*EcoRI* restriction enzyme sites and inserted into the *HindIII*/*EcoRI* sites of pAAV2-SYN-tTAad-BGHpA^15^ to replace the sequence of the improved version of the tetracycline-controlled transactivator (tTAad), resulting in pAAV2-SYN-HBE-BGHpA. An iCre fragment (GenBank AY056050.1) ^42^ was amplified by PCR (primer set P7/P8) from pBOB-CAG-iCRE-SD (#12336, Addgene) and inserted into the *HindIII*/*EcoRI* sites of pAAV2-SYN-HBE-BGHpA, resulting in pAAV2-SYN-iCre-BGHpA.

For construction of pAAV2-SYN-iCre-F2A-EGFP-BGHpA, a *HindIII*-iCre-F2A-*SpeI*-*EcoRI* fragment was amplified by PCR (primer set P7/P9) and inserted into pAAV2-SYN-HBE-BGHpA through the *HindIII*/*EcoRI* sites, resulting in pAAV2-SYN-iCre-F2A-*SpeI*-BGHpA. The EGFP sequence was then amplified by PCR (primer set P10/P11) and inserted into pAAV2-SYN-iCre-F2A-*SpeI*-BGHpA through the *SpeI*/*EcoRI* sites, resulting in pAAV2-SYN-iCre-F2A-EGFP-BGHpA.

We also prepared a helper plasmid containing the sequences of the replication protein of AAV serotype 2 (Rep2) and the capsid protein of AAV-PHP.eB^12^. The following fusion sequence was newly synthesized: (1) nucleotides 146–2202 of the wild-type AAV2 genome (GenBank AF043303.1); (2) nucleotides 1-2229 of the PHP.B capsid protein (GenBank KU056473.1); and (3) nucleotides 4411–4534 of the wild-type AAV2 genome. The fusion sequence was inserted into the *XhoI*/*NotI* sites of pBlueScript II SK (+) (pBSIISK; Stratagene), resulting in pBSIISK-R2CPHP.B. We then performed overlap PCR to introduce the A587D and Q588G mutations in the PHP.B capsid protein using the primer pairs P12/P13, P14/P15, and P12/P15. The resultant PHP.eB capsid fragment (GenBank MF187357.1) was inserted into the *HindIII*/*NotI* sites of pBSIISK-R2CPHP.B to replace the PHP.B sequence, resulting in pBSIISK-R2CPHP.eB.

### Production and purification of AAV vectors

Production and purification of AAV vector particles were performed as reported previously^41,43^. HEK293T cells (RCB2202; Riken Bioresource Center) were incubated in 10 CELLSTAR® cell culture dishes (145 mm) (#639160, Greiner Bio-One). Each genomic plasmid and two helper plasmids, pBSIISK-R2CPHP.eB or pBSIISK-R2C1^15^ and pHelper (28060929; Stratagene) were co-transfected into HEK293T cells using polyethylenimine (23966; Polysciences). Virus particles were collected from the cell lysate and supernatant and purified by ultracentrifugation with OptiPrep (1114542; Axis-Shield). The purified solution was ultrafiltrated and concentrated with Dulbecco’s phosphate-buffered saline (14249-95; Nacalai Tesque) containing 0.001% Pluronic F-68 (24040032; Thermo Fisher Scientific) using an Amicon Ultra-15 (NMWL 30 K; Merck Millipore). Following the ultrafiltration, each virus solution was concentrated to a volume of 100 µL. The virus titer (gc/mL) was measured using quantitative PCR (primer set P16/17) with the Fast SYBR® Green Master Mix (#4385612, Applied Biosystems). We measured the titer of AAV2/PHP.eB-CAG-GFP (#37825; Addgene; 1.0 × 10^13^) as a reference, and the titer was approximately 10.7-fold higher at 1.07 × 10^14^. The following titers were corrected by dividing by 10.7; 2.5 × 10^13^ gc/mL for AAV2/PHP.eB-SynTetOff-EGFP; 9.3 × 10^12^ gc/mL for AAV2/1-SynTetOff-mRFP1; 2.6 × 10^13^ gc/mL for AAV2/PHP.eB-SynTetOff-FLEX-[hChR2-mCherry]; 1.6 × 10^13^ gc/mL for AAV2/1-SYN-iCre-F2A-EGFP-BGHpA; 6.4 × 10^12^ gc/mL for AAV2/1-SYN-iCre-BGHpA; and 4.1 × 10^13^ gc/mL for AAV2/PHP.eB-SYN-GFP-BGHpA^15^. The virus solutions were stored in aliquots at − 80 °C until use.

### Intravenous injection of AAV vectors

Mice were deeply anesthetized with isoflurane (Pfizer Japan). We injected 100 µL of AAV2/PHP.eB vector solutions (diluted 20-fold with saline) into the retro-orbital sinus^44^ or the lateral tail vein^14^. After recovery from anesthesia, the mice were maintained under specific pathogen-free conditions with ad libitum access to food and water for 3–4 weeks.

### Stereotaxic injection of AAV vectors

Mice were deeply anesthetized by intraperitoneal injection of a mixture of medetomidine (0.3 mg/kg; Nippon Zenyaku Kogyo), midazolam (4 mg/kg; Sandoz), and butorphanol (5 mg/kg; Meiji Seika Pharma) and placed in a stereotaxic apparatus. We injected 0.7–2.0 µL of the virus solution into the lateral ventricle (0.5 mm posterior to the bregma, ± 1.0 mm lateral to the midline, 1.8 mm ventral to the brain surface) and/or 0.2 µL into the hippocampal CA2 area (0.9 mm posterior to the bregma, ± 2.3 mm lateral to the midline, 1.9 mm ventral to the brain surface) by pressure through a glass micropipette attached to Picospritzer III (Parker Hannifin). After the surgery, the mice received an intraperitoneal injection of atipamezole (Antisedan; 1.5 mg/kg; Orion) and recovered from anesthesia within approximately 15 min. The mice were maintained under regular health checks for one week after AAV injection.

### Preparation of brain sections

Mice were deeply anesthetized by an intraperitoneal injection of sodium pentobarbital (200 mg/kg; Somnopentyl; Kyoritsu Seiyaku). Mice were then perfused transcardially with 20 mL of phosphate-buffered saline (PBS; pH 7.4) at 20–25 °C, followed by perfusion for 3 min with the same volume of 4% paraformaldehyde (1.04005.1000, Merck Millipore) in 0.1 M phosphate buffer (PB; pH 7.4) at 20–25 °C. The brains were removed and post fixed overnight at 4 °C with the same fixative. After cryoprotection with 30% sucrose in 0.1 M PB, the brains were cut into 40-µm-thick parasagittal, coronal, or horizontal sections on a freezing microtome (SM2000R; Leica Biosystems). Sections were collected in six bottles containing 0.02% sodium azide in PBS and stored at 4 °C until use for free-floating immunostaining.

### Organotypic slice cultures

Whole brains were excised from anesthetized Sprague–Dawley rat pups at P6, and the hippocampi were isolated^21,45,46^. Slices of 400-μm thickness were obtained from the central region of the hippocampi using a McIlwain tissue chopper (Mickle Lab Eng.). The slices were placed on a polytetrafluoroethylene membrane filter (Millicell-CM; Merck Millipore), and the culture medium (see below for composition) was added to the bottom surface of the filter. The prepared cultures were maintained at 37 °C in a humidified atmosphere containing 5% CO_2_. The slice culture medium contained 50% minimal essential medium based on Earle’s salts (21442-25; Nacalai Tesque), 25% Hank’s balanced salt solution (24020-117; Thermo Fisher Scientific), and 25% heat-inactivated horse serum (16050-122; Thermo Fisher Scientific). The culture medium was replaced twice a week with fresh medium throughout the culture period.

For the AAV infection in the culture slices, a mixture of AAV2/1-SynTetOff-mRFP1 and AAV2/PHP.eB-SynTetOff-EGFP was diluted to 1.0 × 10^10^ gc/mL with PBS, and 1 µL of the mixture was added to the culture medium. Forty-eight hours after virus application, the slices were washed with PBS for 3 min and fixed with 4% paraformaldehyde in 0.1 M PB for 4 h at 20–25 °C.

### Whole-cell patch-clamp electrophysiology in acute brain slices

Acute slices were prepared from the hippocampi of juvenile C57BL/6J mice^7,47^. Mice were anesthetized with isoflurane and decapitated. The brains were removed and placed in ice-cold oxygenated (95% O_2_/5% CO_2_) artificial CSF (aCSF) containing (in mM) 127 NaCl, 1.6 KCl, 1.24 KH_2_PO_4_, 1.3 MgSO_4_, 2.4 CaCl_2_, 26 NaHCO_3_, and 10 glucose. The brains were sliced coronally at a thickness of 400 µm using a vibratome (VT1200S; Leica Biosystems) in ice-cold, oxygenated modified aCSF that contained (in mM) 222.1 sucrose, 27 NaHCO_3_, 1.4 NaH_2_PO_4_, 2.5 KCl, 1.0 CaCl_2_, 7.0 MgSO_4_, and 0.5 ascorbic acid^47,48^. Slices were maintained for 30 min at 35 °C in oxygenated aCSF and then incubated for at least 30 min at room temperature before use. All recordings were performed at 33–35 °C. Whole-cell recordings were obtained from mCherry-labeled hippocampal CA2 pyramidal cells using a MultiClamp 700B amplifier and a Digidata 1440 digitizer controlled by pCLAMP10.5 software (Molecular Devices). Glass pipettes (3–6 MΩ) were filled with a solution containing (in mM) 120 K-gluconate, 5.0 KCl, 1.0 MgCl_2_, 10 HEPES, 0.2 EGTA, 10 Na_2_-phosphocreatine, 2.0 MgATP, and 0.1 Na_2_GTP.

For optogenetic manipulation of CA2 pyramidal cells expressing hChR2(H134R)-mCherry, pulses of 465-nm light with a high-power LED illumination system (LEX2-B; BrainVision) were applied for 100 ms through a 40 × objective lens (LUMPlanFl 40x/0.8 W, NA = 0.80; Olympus). The stimulation was repeated ten times.

### Immunofluorescence labeling

All following incubations were performed at 20–25 °C and followed by rinsing twice with PBS containing 0.3% (v/v) Triton X-100 (PBS-X) for 10 min. Free-floating sections (40-µm thick) and slices (400-µm thick) were incubated for 16 h with 1:500-diluted mouse monoclonal antibody against RGS14 (821801; BioLegend), 1:500-diluted mouse monoclonal antibody against STEP (4396S; Cell Signaling Technology), 1:500-diluted rabbit polyclonal antibody against PCP4 (HPA005792; Sigma-Aldrich), 1:100-diluted affinity-purified rabbit antibody against mRFP1^49^, or 1:1000-diluted affinity-purified guinea pig antibody against GFP^50,51^ in PBS-X containing 1% (v/v) donkey serum (S30-100ML; Merck) and 0.12% (w/v) λ-carrageenan (035-09693; Wako Chemicals) (PBS-XCD). The sections and slices were incubated for 4 h with 5 μg/mL Alexa Fluor (AF) 647-conjugated donkey anti-mouse IgG (A31571; Thermo Fisher Scientific), AF568-conjugated goat anti-mouse IgG (A11031; Thermo Fisher Scientific), AF568-conjugated donkey anti-rabbit IgG (A10042; Thermo Fisher Scientific), AF488-conjugated goat anti-guinea pig IgG (A11037; Thermo Fisher Scientific), or AF488-conjugated streptavidin (S11223; Thermo Fisher Scientific) in PBS-XCD, followed by incubation for 30 min with 1:200-diluted NeuroTrace 435/455 Blue Fluorescent Nissl stain (N21479; Thermo Fisher Scientific) in PBS-X. The sections and slices were mounted onto aminopropyltriethoxysilane-coated glass slides (APS-01; Matsunami Glass), and coverslipped with 50% (v/v) glycerol and 2.5% (w/v) triethylenediaminein in 20 mM Tris–HCl (pH 7.6).

### Image acquisition

The sections and slices were observed under a confocal laser scanning microscope (Leica TCS SP8, Leica Microsystems) using 5× air (HCX PL FLUOTAR 5x/0.15, NA = 0.15; Leica Microsystems), 10 × air (HCX PL APO 10x/0.40 CS, NA = 0.40; Leica Microsystems), 16 × multi-immersion (HC FLUOTAR 16x/0.60 IMM CORR VISIR; NA = 0.60; Leica Microsystems), and 25× water-immersion (HC FLUOTAR L 25x/0.95 W VISIR; NA = 0.95; Leica Microsystems) objective lenses with a pinhole at 2–5.6 Airy disk units and a 0.8–1.2 zoom factor. NeuroTrace Blue, EGFP or AF488, AF568, mRFP1, or mCherry, and AF647 were excited using 405-, 488-, 552-, and 638-nm lasers, respectively. Their fluorescence was collected through 410–503, 495–550, 570–650, and 660–750-nm emission prism windows, respectively, and detected using the photon-counting mode of the hybrid detector (HyD; Leica Microsystems).

### Quantification analysis

The density of GFP-expressing cells was determined by counting the cells in regions of 150 µm × 300 µm in the stratum pyramidale of the CA3, CA2, and CA1 regions, and the granule cell layer of the dentate gyrus.

As shown in Fig. S6, the transverse axis was traced along the stratum pyramidale from CA3 to CA1, referring to fluorescent Nissl staining. The location of the putative CA2 region was determined by fluorescent Nissl staining and immunoreactivity for RGS14. The signal intensity in each slice was averaged over 50 pixels, orthogonal to the transverse axis. The x-axis represents the normalized distance from CA3 to CA1 based on the location of the CA2 region.

### Statistics and reproducibility

Data are presented as mean ± standard deviation. The exact values of *n* are indicated in the corresponding Figure legends. For comparisons among independent groups, the Kruskal–Wallis test was used (Figs. 1e and 2d). For comparisons between groups, an unpaired Student’s *t*-test was used (Fig. 2h). All tests were two-sided. Statistical analyses were conducted using MATLAB (Mathworks). Statistical significance was set at *P* < 0.05.

## Supplementary Information

Supplementary Information.

## Data Availability

All data, materials, and custom scripts used in this study are available from the corresponding author on reasonable request.
