# Supplementary material for: Specific AAV2/PHP.eB-mediated gene transduction of CA2 pyramidal cells via injection into the lateral ventricle
Source: Sci Rep. 2023 Jan 6;13:323. doi: 10.1038/s41598-022-27372-8 (PMC9822962; doi:10.1038/s41598-022-27372-8)
Supplement: Supplementary file 1 — Supplementary Information. [file 41598_2022_27372_MOESM1_ESM.pdf]

Supplementary Information

Specific AAV2/PHP.eB-mediated gene transduction of CA2 pyramidal cells via injection into the lateral ventricle

Kazuki Okamoto[1,2,3], Yuji Kamikubo[4], Kenta Yamauchi[1,2,3], Shinichiro Okamoto[1,2,3], Megumu Takahashi[1,2,5,6], Yoko Ishida[1,2,3], Masato Koike[2], Yuji Ikegaya[7,8,9], Takashi Sakurai[4], Hiroyuki Hioki[1,2,10,*]

[1]Department of Neuroanatomy, Juntendo University Graduate School of Medicine, Bunkyo-Ku, Tokyo 113-8421, Japan
[2]Department of Cell Biology and Neuroscience, Juntendo University Graduate School of Medicine, Bunkyo-Ku, Tokyo 113-8421, Japan
[3]Juntendo Advanced Research Institute for Health Science, Juntendo University, Bunkyo-Ku, Tokyo 113-8421, Japan
[4]Department of Cellular and Molecular Pharmacology, Juntendo University Graduate School of Medicine, Bunkyo-Ku, Tokyo 113-8421, Japan
[5]Department of Neuroscience, Graduate School of Medicine, Kyoto University, Kyoto, Kyoto 606-8501, Japan
[6]Research Fellow of Japan Society for the Promotion of Science (JSPS), Chiyoda-ku, Tokyo 102-0083, Japan
[7]Laboratory of Chemical Pharmacology, Graduate School of Pharmaceutical Sciences, The University of Tokyo, Bunkyo-ku, Tokyo 113-0033, Japan
[8]Center for Information and Neural Networks, National Institute of Information and Communications Technology, Suita, Osaka 565-0871, Japan
[9]Institute for AI and Beyond, The University of Tokyo, Bunkyo-ku, Tokyo 113-0033, Japan
[10]Department of Multi-Scale Brain Structure Imaging, Juntendo University Graduate School of Medicine, Bunkyo-Ku, Tokyo 113-8421, Japan

[*]**Correspondence**: Hiroyuki Hioki, M.D., Ph.D., h-hioki@juntendo.ac.jp

Supplementary figures

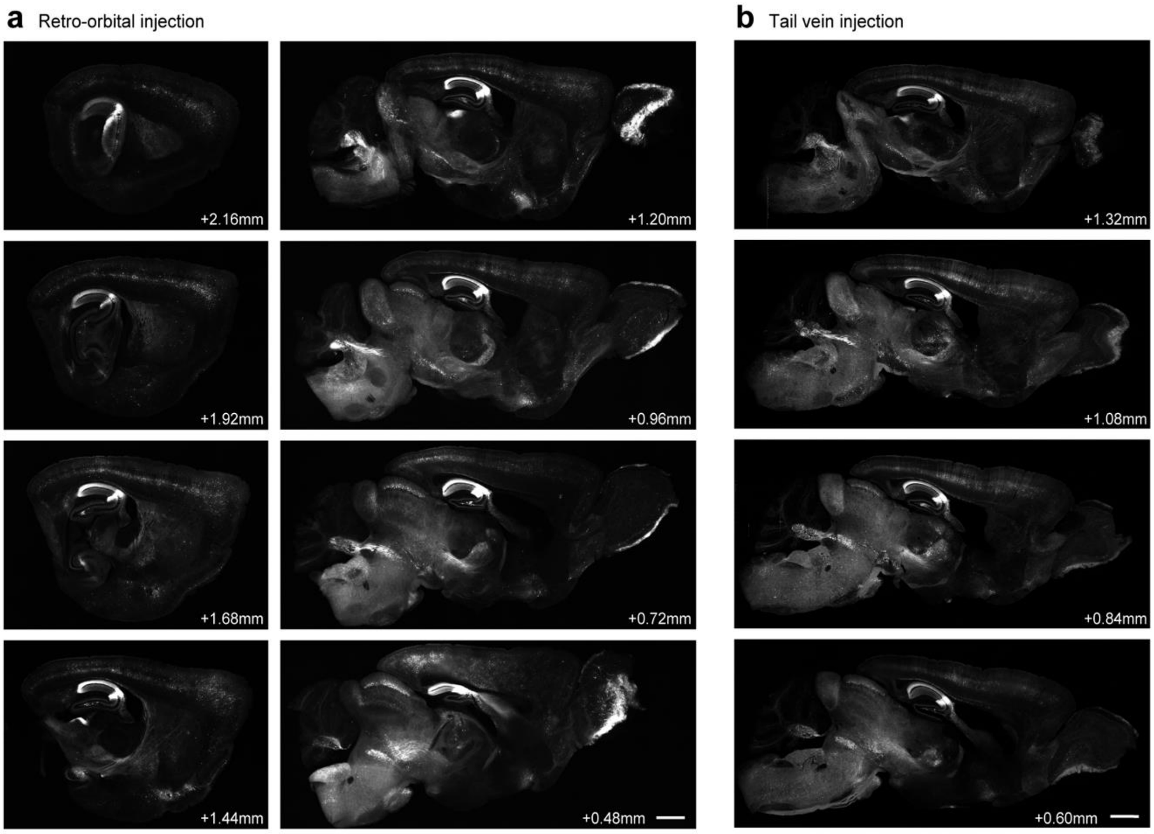

Supplemental figure 1. Whole brain infection with intravenous injection of AAV-PHP.eB. a) GFP expression in sagittal sections after retro-orbital injection of AAV2/PHP.eB-SynTetOff-EGFP vector. Scale bar, 1 mm. **b)** GFP expression in sagittal sections after lateral tail vein injection of the vector. Scale bar, 1 mm.

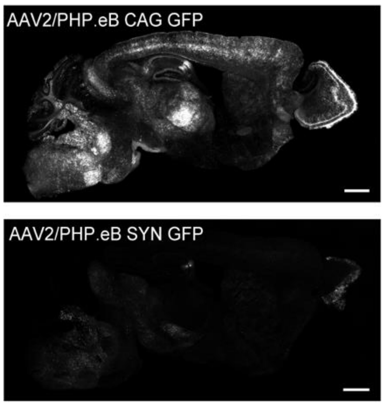

Supplemental figure 2. Differential expression patterns of GFP with CAG or SYN promoters. Strong GFP expression was observed not only in the CA2 but also in the entire brain regions with the CAG promoter, whereas weak GFP expression with the SYN promoter was observed only in the localized regions such as the CA2, olfactory bulb, and cerebellum. Scale bar, 1 mm.

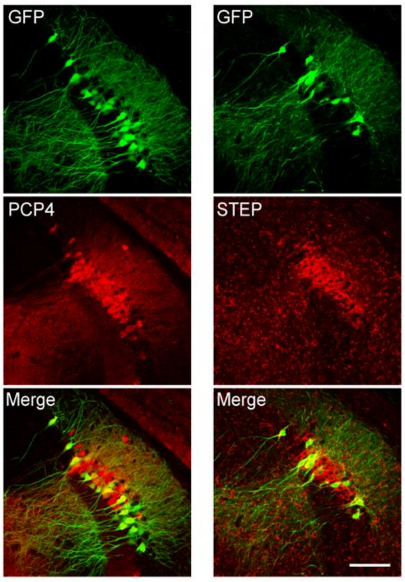

Supplementary figure 3. Immunoreactivity of GFP-expressing cells for PCP4 and STEP. Four weeks after the retro-orbital injection of the AAV2/PHP.eB-SynTetOff-EGFP vector, GFP-expressing neurons showed immunoreactivity for both PCP4 and STEP. Scale bar, 100 μm.

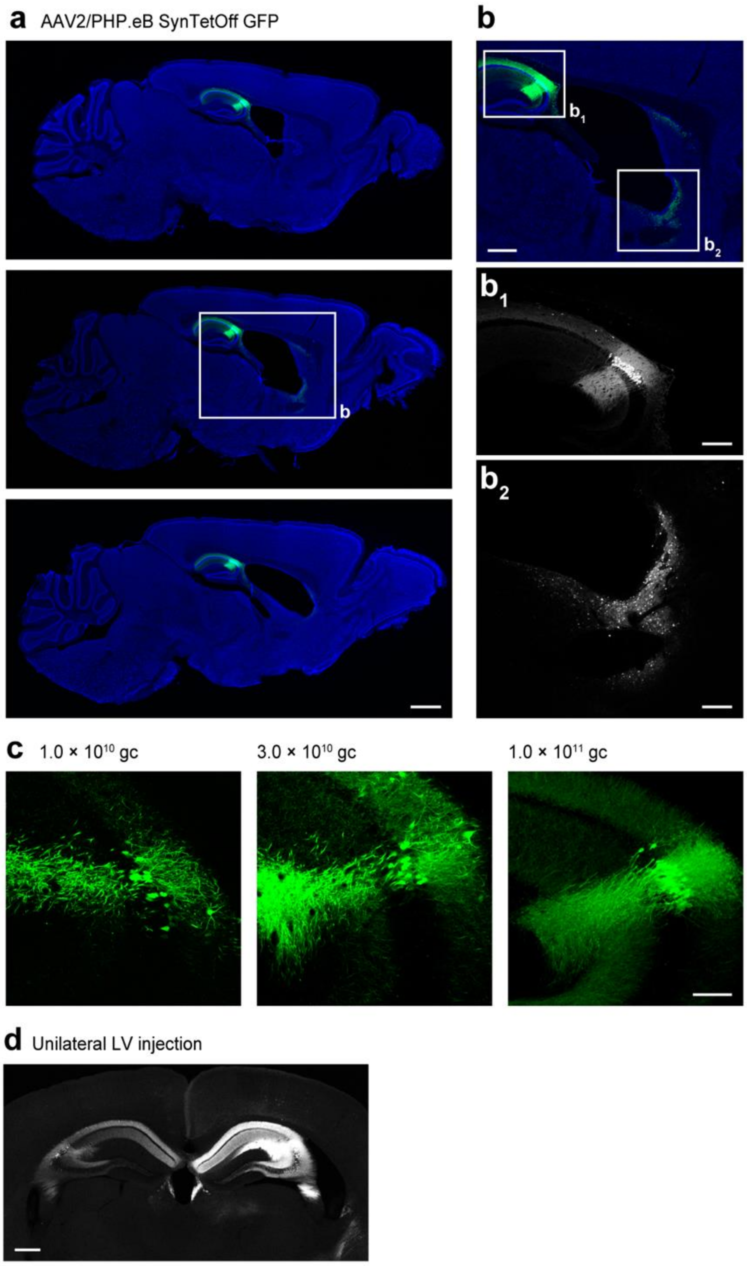

Supplementary figure 4. Restricted infection after LV injection. a) GFP expression in sagittal sections after LV injection of the vector (3.0×10^{10} gc). The sections were labeled with NeuroTrace 435/455 Nissl stain (*blue*). Scale bar, 1 mm. **b)** Enlarged view of the LV region in **a**. Scale bar, 500 µm (b), 200 µm (b_1, b_2). **c)** Representative images of GFP expression in the CA2 pyramidal cells in each virus dose. Scale bar, 100 µm. **d)** Unilateral LV injection. After unilateral LV injection (right side), GFP expression was mainly observed in the ipsilateral CA2 region. Scale bar, 200 µm.

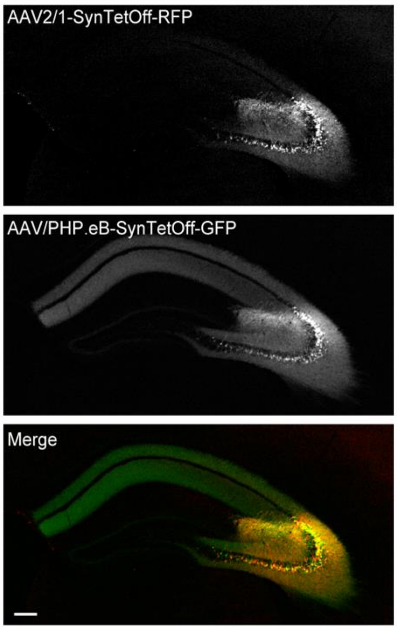

Supplementary figure 5. Direct injection into the CA2 region. Stereotaxic injection of a mixture of AAV2/1-SynTetOff-mRFP1 and AAV2/PHP.eB-SynTetOff-EGFP into the CA2 region. After immunostaining for EGFP and mRFP1, both immunoreactivities were observed not only in CA2 abut also CA3. Scale bar, 200 µm.

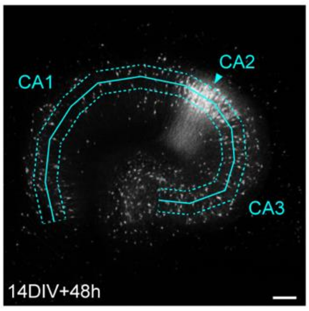

Supplementary figure 6. The transverse axis in cultured hippocampus. Representative image of the transverse axis in the cultured hippocampus. The area surrounded with dot lines was used to calculate the signal strength of RFP, GFP, and RGS14 immunoreactivity. Scale bar, 200 μm.

Supplementary table

P1	5′-AAAAGGATCC**GCCACC**ATGGCCTCCTCCGA-3′
P2	5′-TTTTACGCGTTTAGGCGCCGGTGGAGTGGC-3′
P3	5′-AAAAGTCGAC**GCCACC**ATGGACTATGGCGGC-3′
P4	5′-TTTTGAATTCTTACTTGTACAGCTCGTCCA-3′
P5	5′-AGCTTAGATCTG-3′
P6	5′-AATTCAGATCTA-3′
P7	5′-AAAAAAGCTT**GCCACC**ATGGTGCCCAAGAA-3′
P8	5′-TTTTGAATTCTCAGTCCCCATCCTCGAGCA-3′
P9	5′-TTTTGAATTCACTAGTAGGTCCAGGGTTGGACTCCACGTCTCCCGCCAACTTGAGAAGGTCAAAATTCAAAGTCTGTTTCACTCCGCTTCCGTCCCCATCCTCGAGCAGCC-3′
P10	5′-AAAAACTAGTATGGTGAGCAAGGGCGAGGA-3′
P11	5′-TTTTGAATTCTTACTTGTACAGCTCGTCCA-3′
P12	5′-AAAAAAGCTTCGATCAACTACGCA-3′
P13	5′-AAAGTCCCATCACTCTGGTGGTTTGTGGCC-3′
P14	5′-GAGTGATGGGACTTTGGCGGTGCCTTTTAA-3′
P15	5′-TTTTGCGGCCGCGATATCTGTAGTTAA-3′
P16	5’-GGAACCCCTAGTGATGGAGTT-3’
P17	5’-CGGCCTCAGTGAGCGA-3’

Supplementary table 1. Primers and oligonucleotides used in the present study. Bold characters indicate the Kozak consensus sequence. Underlined sequences indicate F2A sequence.
